# Supplementary material for: Maximizing genetic representation in seed collections from populations of self and cross-pollinated banana wild relatives
Source: BMC Plant Biol. 2021 Sep 9;21:415. doi: 10.1186/s12870-021-03142-y (PMC8431884; doi:10.1186/s12870-021-03142-y)
Supplement: Supplementary file 1 — Additional file 1: Supplementary Methods. [file 12870_2021_3142_MOESM1_ESM.docx]

**Supporting Information**

Kallow, S. et al. Maximizing genetic representation in seed collections from populations of self and cross-pollinated banana wild relatives. BMC Plant Biology.

**Supplementary Methods**

1. Leaf collecting

For each leaf sample a piece of leaf approximately 3x15cm in size from the middle of the leaf was placed into labelled tea bags, only green leaves were used. Tea bags containing leaves were placed into zip-loc plastic bags containing silica gel in the field. During the collecting mission, silica gel was replenished as required to fully desiccate leaf samples. Dried leaf samples were taken to the laboratory following the field mission for DNA extraction.

1. Seed collecting

From each bunch collected, hands (groups of fruits from the former clusters of flowers subtended by one bract) were removed and numbered according to position - with 1 being at the basal end. Hands were then put in paper bags which were organized in cardboard boxes for storage during the remaining field mission. Hands collected in Bougainville and PNG were transported to Meise Botanic Garden for seed extraction. Seeds collected in Viet Nam were extracted and processed in the laboratory of Plant Resource Center (Ha Noi, Viet Nam). Moreover, seeds were transported within one week of the end of the two-week collecting mission, taking less than a week to complete shipment by airplane. During shipment, temperatures were greater than 0 °C and less than 25 °C. In general fruits were received within three weeks of collecting in the field.

1. Seed processing and storage

Seeds were extracted by peeling the epicarp and crumbling or squashing the endocarp after which the seeds were removed by hand. Excess fruit pulp was removed by washing in running water if necessary. In case fruits were hard, they were soaked in water for 24 h prior to seed extraction. Seeds were dried for at least four months at 15% relative humidity (RH) and ~20 °C, and then placed in cold storage at 15% RH and −20 °C sealed in aluminium envelopes.

1. Seed germination and embryo extraction

Initial tests were carried out with ten seeds per selected hand/ bunch to ascertain viability levels. Additional samples were added as required to ensure that enough samples were available for the study. Seeds were either from 2-3 hands from a bunch or were mixed with all seeds from the bunch (this was for three bunches of *M. acuminata* and all bunches of *M. balbisiana*). An average of 16±5 seed per bunch were successfully germinated and used in this study.

Seeds were germinated by embryo rescue to overcome barriers associated with in vivo germination of banana seeds. Seeds were sterilised by soaking them in 96% ethanol for 3 min, followed by 20 min in 1% NaOCl (diluted commercial bleach 5%), containing 1 drop of detergent per 100 mL. Seeds were then rinsed three times in sterile water. Embryos were extracted from seeds using a sterile forceps and scalpel by making an incision in the seed coat next to the micropyle with the scalpel and by manipulating the seed with scalpel and forceps until the testa split open exposing the endosperm and embryo; embryos were then removed by careful manipulation. Embryos were transferred onto autoclaved half MS medium (Murashige & Skoog 1962) in tubes with the haustorium in contact with medium and the embryonic axis upwards. All procedures were carried out in a laminar flow cabinet. Tubes containing embryos were incubated in the dark at 27°C for 14 days, after which they were put in a growth chamber for an additional 14 days (24 h photo-period, 27°C, 50μE m−2 s−1 illumination provided by 36 W Osram cool-white fluorescent tubes). Seedlings were grown in the growth chamber until approximately 8 cm height. Leaves were then collected from seedlings and placed into liquid nitrogen followed by storage at -80°C. Leaves were then lyophilized.

Due to low seed numbers and anticipated low seed viability, DNA was extracted directly from the embryos of the *M. balbisiana* accessions prior to germination. For this, embryos were extracted as described. Embryos were then washed three times in deionized water to remove any endosperm and then placed in individual tubes (2ml Eppendorf) with 10µl of cetyltrimethylammonium bromide (CTAB) extraction buffer. DNA extraction was then immediately carried out.

1. DNA extraction

Approximately 15mg of lyophilized tissue was used for DNA extraction. Leaves were ground using a stainless-steel ball in each tube. Ground samples were washed using sorbitol incubation for 20 mins on ice (for extraction form embryos this stage was omitted). Lysis was carried out using CTAB 2x buffer and mercapto-ethanol for at least an hour at 60°C. Chloroform/isomylalcohol (24:1) was used for extraction twice. DNA was precipitated using -20°C isopropanol at 80% of the volume of the extract followed by an ethanol (70%) washing step at -20°C. DNA pellets were dissolved in 50µl of TE buffer.

1. PCR
   1. Markers

Taxon specific polymorphic microsatellite markers were used for each species and arranged in multiplexes (Table S1), using the M13 labelling protocol of Schuelke (1). One primer for each pair was extended with one of four M13 (-21) Q-tail that corresponded with Q-tails of one of four fluorescent labels (6-FAM, VIC, NED or PET). For *M. acuminata* a suite of primers was developed from previous studies (2-8). A total of 86 primer pairs were tested for amplification individually. Amplified polymorphic primers were then arranged in a total of 15 multiplexes using Multiplex Manager (9) and Multiple Primer Analyzer (10) and tested with 12 samples for amplification and polymorphism. From this, 20 markers arranged in four multiplexes were selected. For *M. balbisiana,* the multiplex arrangement of Bawin et al. (11), 18 SSR markers organized into four multiplexes was used. For *M. maclayi* s.l., a total of 16 specific SSR markers were developed and optimized by Genoscreen (Lille) and arranged in four multiplexes.

- 1. Amplification and sequencing

Microsatellite regions were amplified using the Type-it Microsatellite PCR Kit (Quigen, Venlo, the Netherlands). We carried out 15µl reactions with 7.5µl of 2x Master-Mix, 1.5µl primer mix (made of 0.7µM of Q-tail primer, 2µM of non-Q-tailed primer and 2µM of each Q-dye), 3µl of Q solution (5x), 2 µl of H2O and 1 µl of DNA. PCR was carried out with 5 minutes at 94°C; 25 cycles of 30 seconds at 94°C, 45 seconds at 57°C and 60 seconds at 72°C; 10 cycles of 30 seconds at 94°C, 45 seconds at 53°C and 60 seconds at 72°C; finally 10 minutes at 72°C. Resultant product was diluted 1/3 prior to sequencing on an ABI 3730 sequencer (Applied Biosystems, Foster City, California, US)*.*

References

1. Schuelke M. An economic method for the fluorescent labelling of PCR fragments. Nature Biotechnology. 2000;18:233-4.

2. Miller RN, Passos MA, Menezes NN, Souza MT, do Carmo Costa MM, Azevedo VCR, et al. Characterization of novel microsatellite markers in Musa acuminata subsp. burmannicoides, var. Calcutta 4. BMC research notes. 2010;3(1):148.

3. Passos M, Cruz V, Emediato F, Teixeira C, Souza M, Matsumoto T, et al. Development of expressed sequence tag and expressed sequence tag-simple sequence repeat marker resources for Musa acuminata. Aob Plants. 2012.

4. Ge XJ, Liu MH, Wang WK, Schaal BA, Chiang TY. Population structure of wild bananas, *Musa balbisiana*, in China determined by SSR fingerprinting and cpDNA PCR-RFLP. Molecular Ecology. 2005;14(4):933-44.

5. Rotchanapreeda T, Wongniam S, Swangpol SC, Chareonsap PP, Sukkaewmanee N, Somana J. Development of SSR markers from *Musa balbisiana* for genetic diversity analysis among Thai bananas. Plant Systematics and Evolution. 2016;302(7):739-61.

6. Lagoda PJL, Dambier D, Grapin A, Baurens FC, Lanaud C, Noyer JL. Nonradioactive sequence-tagged microsatellite site analyses: A method transferable to the tropics. Electrophoresis. 1998;19(2):152-7.

7. Hippolyte I, Bakry F, Seguin M, Gardes L, Rivallan R, Risterucci AM, et al. A saturated SSR/DArT linkage map of Musa acuminata addressing genome rearrangements among bananas. Bmc Plant Biology. 2010;10:18.

8. D'Hont A, Denoeud F, Aury JM, Baurens FC, Carreel F, Garsmeur O, et al. The banana (*Musa acuminata*) genome and the evolution of monocotyledonous plants. Nature. 2012;488(7410):213-20.

9. Holleley CE, Geerts PG. Multiplex Manager 1.0: a crossplatform computer program that plans and optimizes multiplex PCR. Bio Techniques. 2009;46(7):511-7.

10. ThermoFisher. Multiple Primer Analyzer 2020 [Available from: https://www.thermofisher.com/be/en/home/brands/thermo-scientific/molecular-biology/molecular-biology-learning-center/molecular-biology-resource-library/thermo-scientific-web-tools/multiple-primer-analyzer.html.

11. Bawin Y, Panis B, Vanden Abeele S, Li Z, Sardos J, Paofa J, et al. Genetic diversity and core subset selection in ex situ seed collections of the banana crop wild relative Musa balbisiana. Plant Genetic Resources: Characterization and Utilization. 2019;17(6):536-44.
